# Fitness Cost Associated With Enhanced *EPSPS* Gene Copy Number and Glyphosate Resistance in an *Amaranthus tuberculatus* Population

**DOI:** 10.3389/fpls.2021.651381

**Published:** 2021-06-29

**Authors:** Helen M. Cockerton, Shiv S. Kaundun, Lieselot Nguyen, Sarah Jane Hutchings, Richard P. Dale, Anushka Howell, Paul Neve

**Affiliations:** ^1^NIAB EMR, Kent, United Kingdom; ^2^Warwick Crop Centre, The University of Warwick Wellesbourne, Warwick, United Kingdom; ^3^Syngenta, Jealott’s Hill International Research Centre, Bracknell, United Kingdom; ^4^Rothamsted Research, Harpenden, United Kingdom; ^5^Department of Plant and Environmental Sciences, University of Copenhagen, Tåstrup, Denmark

**Keywords:** evolution, fitness, herbicide resistance, resource competition, trade-off

## Abstract

The evolution of resistance to pesticides in agricultural systems provides an opportunity to study the fitness costs and benefits of novel adaptive traits. Here, we studied a population of *Amaranthus tuberculatus* (common waterhemp), which has evolved resistance to glyphosate. The growth and fitness of seed families with contrasting levels of glyphosate resistance was assessed in the absence of glyphosate to determine their ability to compete for resources under intra- and interspecific competition. We identified a positive correlation between the level of glyphosate resistance and gene copy number for the 5-enolpyruvylshikimate-3-phosphate synthase (EPSPS) glyphosate target, thus identifying gene amplification as the mechanism of resistance within the population. Resistant *A. tuberculatus* plants were found to have a lower competitive response when compared to the susceptible phenotypes with 2.76 glyphosate resistant plants being required to have an equal competitive effect as a single susceptible plant. A growth trade-off was associated with the gene amplification mechanism under intra-phenotypic competition where 20 extra gene copies were associated with a 26.5 % reduction in dry biomass. Interestingly, this growth trade-off was mitigated when assessed under interspecific competition from maize.

## Introduction

Crop protection practices are designed to limit the incidence and impacts of crop pests, including weeds. Unfortunately, these practices also provide a selection pressure for pest organisms to overcome control strategies. Pathogens, pests and weeds have all evolved resistance to pesticides through selection of *de novo* mutations and/or standing genetic variation or by immigration of pollen or seed from external populations (Hawkins et al., 2018; Molin et al., 2020). Evolutionary theory states that adaptation to resist or tolerate a novel stress may incur a fitness cost when the individual is returned to the original “stress-free” environment (Herms and Mattson, 1992; Bergelson and Purrington, 1996; Coustau et al., 2000; Fry, 2003; Vila-Aiub et al., 2011, 2015). Ultimately, the quantification of fitness costs in a herbicide resistant population can inform management strategies to reduce the equilibrium frequency of resistant genotypes in order to assist effective weed control (Cousens and Mortimer, 1995; Jordan, 1999; Vila-Aiub et al., 2005a; Vila-Aiub, 2019).

The size of a fitness benefit in the presence of the herbicide determines the rate at which a resistance allele will establish within a population under selection (Roux et al., 2006), whereas the presence of a resistance cost determines if the frequency of a resistance allele will reduce in the absence of selection (Coustau et al., 2000). The benefit-cost fitness balance is thought to determine the frequency of a resistance allele under different agricultural management regimes (Roux et al., 2006; Vila-Aiub et al., 2011).

The fitness cost associated with an adaptive allele may manifest as a direct cost as a result of a pleiotropic effect of the resistance allele, or *via* ecological trade-offs in life history traits such as biomass (McCloskey and Holt, 1990), emergence date (Vila-Aiub et al., 2005b), height and flowering time (Roux et al., 2005) each of which may ultimately result in a direct fitness cost within a resource limited environment (Délye et al., 2013). Fitness costs have been widely reported for antibiotic resistance (Lee and Edlin, 1985; Nguyen et al., 1989; Bentley et al., 1990; Purrington and Bergelson, 1997). However, such costs are not universally observed for herbicide resistant weed populations, and where costs have been observed they have been shown to be influenced by the genetic background of the population (Paris et al., 2008; Vila-Aiub et al., 2009b). It is believed that fitness costs, if present, can be magnified by intra and inter-specific competition for resources (Bergelson and Purrington, 1996).

The mechanism of evolved resistance may also influence the magnitude and type of fitness cost that is expressed (Paris et al., 2008). For example, resource allocation theory states that a resistant individual which metabolises xenobiotics will divert resources away from growth and reproduction and toward defense, resulting in a cost of adaptation (Bergelson and Purrington, 1996). Furthermore, additional gene copy numbers may be associated with costs due to allocation of resources toward over-production of an amplified protein (Tang and Amon, 2013). A mutation in a pesticide target enzyme may impact on the efficacy of enzyme function (Vila-Aiub et al., 2005b; Tardif et al., 2006; Menchari et al., 2007; Vila-Aiub et al., 2019), leading to fitness costs through the disruption of normal metabolic processes. To date, there has been a diverse range of glyphosate resistance mechanisms that have evolved in agricultural weed populations (Gaines et al., 2019). Resistance mechanisms include nucleotide polymorphisms in the target site 5-enolpyruvylshikimate-3-phosphate synthase (*EPSPS*) (Perotti et al., 2019), enhanced *EPSPS* numbers (Gaines et al., 2010), vacuole sequestration (Ge et al., 2012) and rapid cell death (Moretti et al., 2018; van Horn et al., 2018).

*Amaranthus tuberculatus* (Common Waterhemp) is a prevalent, problematic weed in cropping systems in the United States and Canada. Over the last three decades genetically modified glyphosate tolerant crops have been grown extensively in those systems, resulting in multiple annual applications of glyphosate. Indeed, the major advantage of glyphosate tolerant crops is the provision of a simple weed management strategy. A prolonged reliance on glyphosate tolerant crops has provided the strong selection pressure required for the evolution of glyphosate resistant weed populations (Neve, 2008). By 1998, variable glyphosate responses had been identified in *A. tuberculatus* field populations (Zelaya and Owen, 2002, 2005), but it was 10 years before the first confirmed incidence of a field-evolved glyphosate resistant population (Legleiter and Bradley, 2008). Today, glyphosate resistant *A. tuberculatus* has been documented in 18 states of the United States and in Ontario, Canada (Heap, 2021). Canadian populations have established from multiple origins, both through gene flow from bordering epidemics and through *de novo* evolution of resistance (Kreiner et al., 2019).

Here we studied a glyphosate resistant population of *A. tuberculatus* to establish the mechanism of glyphosate resistance and the presence of fitness costs associated with that mechanism. We test the hypothesis that resistance is associated with a fitness cost in the absence of glyphosate by carrying out two competition experiments. A response surface experiment was used to compare the competitive response and effect of resistant and susceptible phenotypes. Whereas a neighborhood design experiment was used to determine if there was a trade-off between seed family resistance level and resistance cost under intra and inter specific competition with maize.

## Materials and Methods

### Plant Material

**FIGURE 1:**
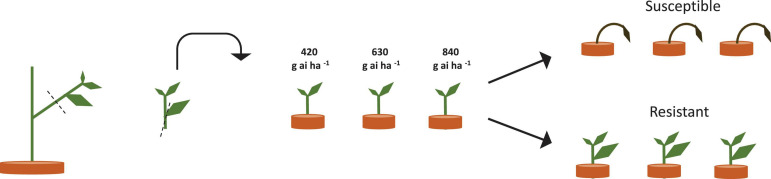
Scheme of clone propagation method for parental plant herbicide phenotyping. Three clonal leaf cuttings were removed from individual parental plants and transplanted into pots to encourage rooting and re-growth. 18 days after propagation, three established clones per plant were treated with 420, 640, or 840 g ai ha^–1^ of glyphosate. Survival and growth were assessed, and plants were scored as resistant or susceptible depending on plant survival, mortality and glyphosate symptoms at the three glyphosate doses.

*Amaranthus tuberculatus* seed was collected from glyphosate-resistant soybean fields (N 44.78, W 95.21) in Renville, Minnesota, United States. Original seed collection was performed in 2007 after four glyphosate applications resulted in poor weed control. The generation of resistant and susceptible seed families from a single population permits fitness parameters to be studied in the genetic background in which resistance has evolved (Vila-Aiub et al., 2011). Resistant seed families were generated through crossing 10 resistant female *A. tuberculatus* plants with 10 resistant male *A. tuberculatus* plants in a bulk cross, susceptible seed families were generated in a similar fashion by crossing susceptible individuals. The parental resistant and susceptible *A. tuberculatus* plants were identified by phenotyping vegetative clones as outlined in Figure 1. After cross pollination and seed maturation, seed collected from a single plant formed a seed family. A subsequent dose response experiment confirmed resistance levels of seed families and six families were selected for subsequent experiments. These families encompassed a range of glyphosate resistance levels. The dose response experiment was conducted in May–July 2011, whilst subsequent experiments were conducted in June–Sept 2012.

### Seed Family Dose Response Resistance Quantification

*Amaranthus tuberculatus* seed from six glyphosate-selected seed families was imbibed on Levington growing media (FS2) at 5°C for 7 days. and germinated in a glasshouse at 24:18°C, 16:8 h (day: night), with supplementary lighting. Seedlings were transplanted 14 days post germination into 1.5 L pots (10 seedlings per pot) containing medium grade sphagnum moss peat and pots were maintained in the glasshouse. Commercial glyphosate (touchdown IQ) was applied at the 6–8 leaf stage at 0, 105, 210, 420, 840, or 1,680 g ai ha^–1^ using a Berthoud Velmorel 200 pro knapsack sprayer and Deflector Anvil Polijet nozzle (D/1.2/1) at 200 kPa, 2 km h^–1^ at 40 cm above the plant canopy at a water volume of 300 L ha^–1^. Six seed families were assessed across 30 replicate plants per treatment, with 10 seedling in each of 3 replicate pots and arranged in a randomized block design. A susceptible field population was used as a glyphosate sensitive control, this population was sourced from Azlin Seed Service and had a similar morphology to the resistant populations. Percentage survival and above ground biomass was assessed 21 days after glyphosate treatment. Mortality was assigned based on observation of complete necrosis, apical meristem necrosis, and root system disintegration. The lethal dose required to kill 50% of individuals (LD_50_) was determined for each seed family based on the best fitting dose response model (log logistic, Weibull 1 or Weibull 2) where goodness-of fit was determined using a Pearsons’s chi squared test. Analysis was conducted in R (R version 2.15.1, R Core Team, 2009) using the drc package (Ritz and Streibig, 2005). LD_50_ comparisons between seed families were conducted through using a student’s *t*-test. Resistance indices (RI) were calculated as the LD_50_ of the putative resistant population as a ratio of the standard sensitive LD_50_ (LD_50_R / LD_50_S).

### Resistance Mechanism Determination

Elucidating the mechanism of resistance present in a population provides genotypic context for phenotypic observations. Sequencing of the gene for the glyphosate target enzyme (EPSPS) and determination of glyphosate translocation (for seed family 153 and 315) were carried out as detailed in the Supplementary Methods. EPSPS gene copy number (GCN) quantification was assessed through qPCR as detailed below.

DNA extraction was achieved through grinding approximately 0.5 g of *A. tuberculatus* leaf material in a pestle and mortar with 3 ml of grinding buffer (100 mM NaOAc pH 4.8; 50 mM EDTA pH8; 500 mM NaCl; 2% PVP; 1.4% SDS; H_2_O) and incubated at 65 °C for 15 min, 1 ml of ammonium acetate was added to the supernatant and incubated at 65 °C for 10 min. Polysaccharide contaminants were removed by two subsequent additions of phenol: chloroform: iso-amyl alcohol (25:24:1) (pH 8) and one chloroform: iso-amyl alcohol (24:1). After each addition, solutions were mixed by inversion and centrifuged at 13,000 rpm for 5 min and the aqueous layer was kept. Precipitation of DNA was achieved through addition of 0.6 volume of cold isopropanol and 0.1 volume of 3 M NaOAc, chilled at −20°C for 30 min and centrifuged at 13,000 rpm for 10 min. Supernatant was discarded, pellets were dried for 10 min and DNA was resuspended in 10 mM Tris–HCl (Ph8) 1 mM EDTA.

*EPSPS* gene copy number was determined by qPCR through measuring the relative quantity of *EPSPS* to housekeeping gene amplicons, in genomic DNA. Master mixes of Takyon Master Mix for SYBR^®^ Assay mix (Eurogentec): Water: Forward primer: Reverse primer: DNA template (5:1:1:2:1.25). Primer sequences in Supplementary Table 2 for the gene of interest *EPSPS* and housekeeping genes carbamoyl phosphate synthetase () and acetolactate synthase (*ALS*). Reactions were run in triplicate for 6–8 individuals per seed family on an Applied Biosystems^®^ 7500 Fast Real-Time PCR System. The individuals that were assessed to determine seed family gene copy number (GCN) were siblings of the plants used in the competition experiments. qPCR cycle conditions were 95°C for 3 min, 40 cycles of 95°C for 3 s, 60°C for 40 s. GCN was calculated by Eq. 1 and2.

(1) $$G\,C\,N=2^{-△\,△\,\text{CT}}$$

(2) $$△\,△\,\text{CT}=\left({\bar{x}}_{E\,C\,T}-{\bar{x}}_{A\,C\,C\,T}\right)-\left({\bar{y}}_{E\,C\,T}-{\bar{y}}_{C\,A\,C\,T}\right)$$

Where CT is the cycle threshold of *EPSPS* (E) or a combination of the control genes *CPS* and *ALS* (CA) and $\bar{y}$ represents the standard sensitive geometric mean and $\bar{x}$ represents the seed family geometric mean. A regression analysis was conducted to determine the relationship between the average seed family GCN and whole plant glyphosate LD_50_ and an ANOVA and Tukey *post hoc* test was used to assess whether GCN varied between seed family.

### Response Surface Competition Experiment

**FIGURE 2:**
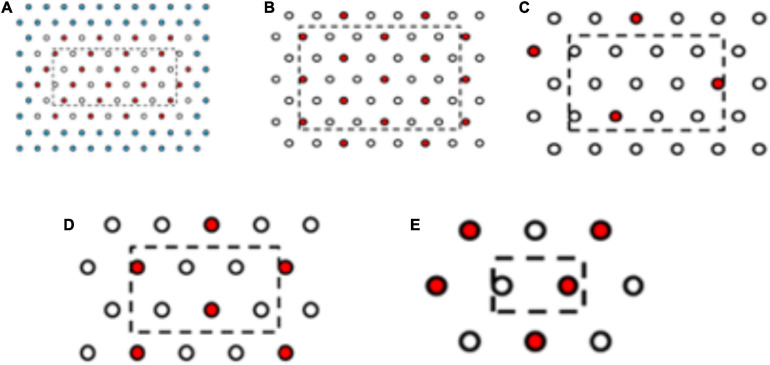
Planting arrangements at selected densities and proportions of resistant and susceptible seed families. **(A)** R:S, 3:3 plant ratio, 1,100 plants m^–2^, **(B)** R:S 4:2, 560 plants m^–2,^ **(C)** R:S 6:1, 300 plants m^–2^, **(D)** R:S 4:2 200 plants m^–2^, **(E)** R:S 3:3 100 plants m^–2^. Red and white spots represent the plant position for plants from the most resistant and the most susceptible seed family phenotypes are interchangeable. Three replicate boxes were created for density **(A–D)** and six replicate boxes for density e. Due to the high seedling volume required for the 1,100 plants m^–2^ treatment, two rows of spacer plants (blue) were added to the outside of the layout, spacer plants were from seed family 153. Plants within the dashed line box were harvested, plants outside of the box acted as edge effect plants.

Plants from the more resistant seed family “9” (RI = 4.8) and the more susceptible seed family “29” (RI = 1.7) were transplanted in planting boxes in a triangular grid at five densities of 1,100, 560, 300, 200, and 100 plants m^–2^ across seven resistant: susceptible proportions 6:0, 5:1, 4:2, 3:3, 2:4, 1:5, and 0:6 (Figure 2). Rather than conducting a full factorial design representing all proportions and density combinations, 19 treatments were selected for study (Table 1). Three replicate planting boxes were produced per treatment (six replicates for 100 plants m^–2^ treatment) giving a total of 66 boxes arranged in a randomized block design. Plants were transplanted into boxes containing 13 kg of a 2:1 mix of top soil: medium grade sphagnum moss peat (pH = 7.6, *K* = 176.3, *P* = 80.4, NO_3_ = 146.6, mg = 377.9 μg g^–1^). Border plants were included to eliminate edge effects but were excluded from the analysis.

**TABLE 1:** Plant densities and resistant: susceptible (R:S) proportions used for the response surface experiment.

	**Density (plants m^–2^)**				
**R:S**	**1,100**	**560**	**300**	**200**	**100**
6:0	+	+	+	+	+
5:1			+		
4:2		+		+	
3:3	+		+		+
2:4		+		+	
1:5			+		
0:6	+	+	+	+	+

*+ marks selected conditions.*

Above ground dry biomass were recorded at 114 days after transplanting. Log transformed biomass data was analyzed in R (R version 2.15.1: 2012-06-22) (R Core Team, 2009) using the nonlinear least squares (nls) function in the stats package version 3.0.1. A hyperbolic curve (Pedersen et al., 2007, Equation 3) described the relationship between plant biomass, density and proportion and allowed the calculation of *a*, *b* and *c* parameters, where *a* is maximum plant biomass in the absence of competition, *b* is the competitive response and *c* is the relative competitive effect of phenotypes.

(3) $$\log\left(y^{i}\right)=l\,o\,g\,\left(\frac{a}{1+b_{i}\,\left(N_{i}+c_{i\,j}\,N_{j}\right)}\right)+\varepsilon$$

Effective density was calculated as *N*_*i*_ + *c_*ij*_N_*j*_* where *N* is the density of the *i* or *j* phenotype and *c*_*ij*_ is the *c* parameter determined by the either the *i* or *j* specific parameter. Parameters were compared using z-tests. Parameter *c* was tested for a difference from 1 using a *T*-test. Analysis included plants that had died as a result of competition.

### Neighborhood Design Competition Experiment

**FIGURE 3:**
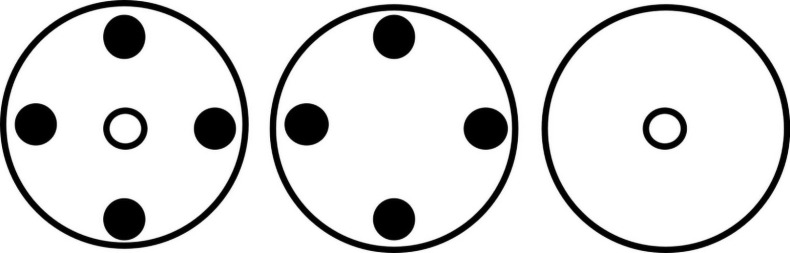
Layout of plants in neighborhood design experiment. Large circles represent pots. Filled circles represent *A. tuberculatus* plants and open small circles represent maize plants.

Maize (cv. Kangaroo) and *A. tuberculatus* seedlings were transplanted as indicated in Figure 3, into 4 L pots containing 2 kg of the growth medium outlined above. Weed seedlings were transplanted so that cotyledons were 2 cm above the soil surface at the V1 corn growth stage. The three treatments denoted in Figure 3 allowed the investigation of the impact of *A. tuberculatus* on maize (*a* and *c*) and the impact of maize on *A. tuberculatus* (*a* and *b*). Layouts *a* and *b* are produced for the six *A. tuberculatus* seed families (1,29, 90, 141, 153, and 315) with contrasting levels of glyphosate resistance. In this way, layout *a* can determine the trade-off between plant growth and seed family resistance levels under interspecific competition and layout *b* can be used to assess this relationship under intraspecific competition. Replicate pots were arranged in a randomized block design, with ten replicates for each treatment, apart from maize-alone (*c*) which had 20 replicates. Competition experiments were conducted in a polytunnel from June to September. Total above ground and reproductive fresh biomass of maize and *A. tuberculatus* was recorded 107 days after transplanting. The overall impact of interspecies competition and biomass allocation was assessed through *T*-tests. Pearson correlations were calculated between biomass traits and resistance metrics to determine trade-offs between fitness and resistance.

## Results

### Quantitative Segregation of Glyphosate Resistance

Dose response experiments were used to assess the herbicide resistance status of the A. tuberculatus field-collected population and the derived seed families. The field-collected population had a glyphosate resistance index (RI) of 3.2. Derived seed families had glyphosate RIs ranging from 1.7 to 5.3 (Table 2). The wide range of resistance phenotypes (levels of resistance) observed within and amongst seed families are suggestive of inheritance of a quantitative resistance trait. The LD_50_ for each seed family was significantly different from all but neighboring seed families (Supplementary Table 2). The variation in population-level resistance estimates provides a good range of seed families with which to test a trade-off between resistance level and fitness cost.

**TABLE 2:** Seed family (SF) Gene copy number (GCN) of 5-enolpyruvylshikimate-3-phosphate synthase (*EPSPS*) relative to carbamoyl phosphate synthetase (*CPS*) and acetolactate synthase (*ALS*).

**SF**	**GCN**	**LD_50_**	**RI**	**Model**
1	11.6 (± 5.5)^a^	882 (± 97)	5.3 (± 1.3)	W1
29	1.2 (± 0.8)^b^	335 (± 40)	1.7 (± 0.7)	W1
90	3.8 (± 1.2)^b^	648 (± 80)	3.3 (± 0.9)	W1
141	0.9 (± 0.1)^b^	457 (± 37)	2.3 (± 0.7)	W1
153	2.1 (± 4.9)^ab^	401 (± 53)	1.9 (± 0.7)	LL
315	7.2 (± 1.4)^ab^	620 (± 66)	3.5 (± 0.9)	W1

*6–8 plants were assessed for GCN per seed family.*

*Superscript letters denote significance groupings for GCN based on an ANOVA and Tukey *post hoc* test.*

*Lethal dose required to kill 50 percent of individuals (LD_50_) calculated on 30 replicate plants per treatment.*

*Resistance Index (RI) relative to the field sensitive population with an LD_50_ of 207.21 (±20.2).*

*Standard errors are presented in parentheses.*

*Model represents the best fitting dose response model; either log logistic (LL) or Weibull’s 1 (W1).*

### Gene Amplification Correlated With Resistance to Glyphosate

**FIGURE 4:**
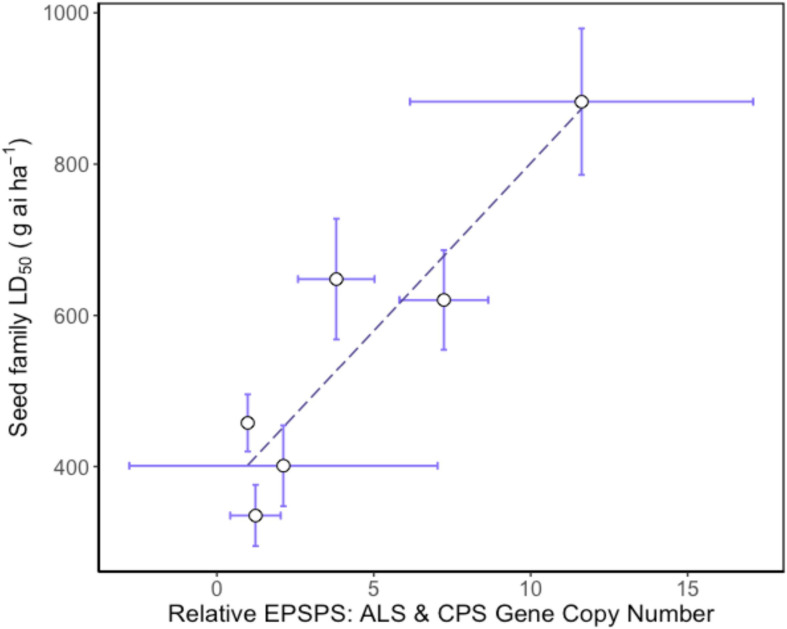
Relationship between relative mean *EPSPS* gene copy number and glyphosate resistance phenotype in *Amaranthus tuberculatus* seed families. Mean 5-enolpyruvylshikimate-3-phosphate synthase (*EPSPS*) gene copy number is relative to carbamoyl phosphate synthetase (*CPS*) *and* acetolactate synthase (*ALS*) reference genes. The LD_50_ for each seed family was calculated using 30 replicate plants for each of six glyphosate doses and 6–8 replicate plants from each seed family were assessed for *EPSPS* gene copy number. Regression analysis: *f*-statistic = 22.99; *df* = 1; 4, *p* = 0.0087, *R*^2^ = 0.85. Error bars are standard errors of the mean.

Higher *EPSPS* gene copy numbers were present in resistant seed families with mean values ranging between 3.8 and 11.6 copies. Gene copy numbers varied between seed family with one individual in seed family 153 exhibiting a high GCN, leading to a relatively large standard error (Table 2). There was a significant positive relationship between average seed family *EPSPS* relative gene copy number and seed family LD_50_ (Figure 4) (f-statistic = 22.99; df = 1; 4, *p* = 0.0087, *R*^2^ = 0.85). The linear model estimates indicate that each gene copy increased glyphosate LD_50_ by 44.3 g ai ha^–1^. Increased *EPSPS* gene copy number was associated with enhanced glyphosate resistance in the *A. tuberculatus* Renville population. Target site sequencing of the *EPSPS* gene confirmed the absence of non-synonymous mutations in the active site of resistant individuals (data not shown). Furthermore, there was no significant impairment of translocation of ^14^C labeled glyphosate away from the site of application in resistant individuals (Supplementary Figure 1).

### Decreased Competitive Effect of Glyphosate Resistant Plants

**FIGURE 5:**
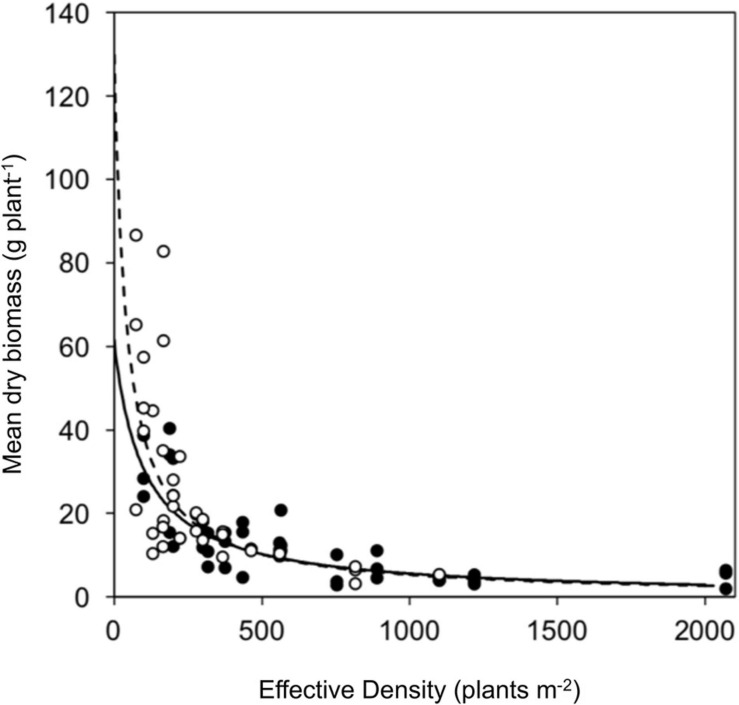
Vegetative growth of a glyphosate sensitive and resistant seed family in response surface competition experiment. Symbols represent mean biomass of resistant (black) and susceptible (white) phenotypes from replicate planting boxes at each effective density. Curves show fitted hyperbolic models for resistant (solid) and the susceptible (dashed) phenotypes.

Competitive effect and response were measured using a response surface experiment. No significant difference was observed between resistant (seed family 9) and susceptible (seed family 29) plant biomass in the absence of competition (parameter *a*) nor in the response to competition (parameter *b*). However, the competitive effect on neighboring plants was greater for susceptible plants (parameter *c*; Z_39_ = 3.107, *p* < 0.001). Resistant plants had a reduced competitive effect when compared to susceptible plants, where a substitution rate of 2.76 was determined for resistant phenotypes and 0.48 for susceptible phenotypes. Explicitly, 0.48 susceptible plants are required to have an equal competitive effect to that of a single resistant plant and 2.76 resistant plants are required to have an equal competitive effect to a single susceptible plant. The relative competition dynamics of resistant and susceptible phenotypes are shown in Figure 5 and Table 3.

**TABLE 3:** Hyperbolic model parameters for resistant (R) and susceptible (S) phenotypes.

**Phenotype**	**a parameter**	**b parameter**	**c parameter**
R	61.5 (30.42)	0.0100 (0.0066)	2.76 (0.710)
S	129.7 (84.02)	0.0235 (0.0177)	0.48 (0.181)

**a* indicates the maximum yield in the absence of competition; *b* indicates the competitive response of a phenotype to an increase in density and *c* denotes the relative competitive effect of the phenotype.*

*Three replicate boxes were created for densities of 1,100, 560, 300, and 200 plants m^–2^ and six replicate boxes for density 100 plants m^–2^.*

*No significant difference was found between R and S for parameter *a* (biomass without competition) and *b* (competitive response) but parameter *c* (competitive effect) was greater for S plants; Z_39_ = 3.107, *p* < 0.001.*

*Values in brackets are standard errors of the mean.*

### Interspecific Competition Influences Resource Allocation

Overall competition between the maize and *A. tuberculatus* seed families was assessed; Four *A. tuberculatus* plants reduced the above-ground biomass of one maize plant by 42% (*t*_78_ = −0.07, *p* < 0.001) whereas maize reduced the above ground biomass of *A. tuberculatus* by 37% (*t*_118_ = −8.24, *p* < 0.001). *A. tuberculatus* seed families with contrasting resistance levels were all observed to consistently divert resources away from vegetative growth in order to allow 10% more resource allocation toward reproduction in the presence of maize (*t*_118_ = −7.44, *p* < 0.001). This resource allocation was observed across all seed families.

### Trade-Off Associated With Resistance

**FIGURE 6:**
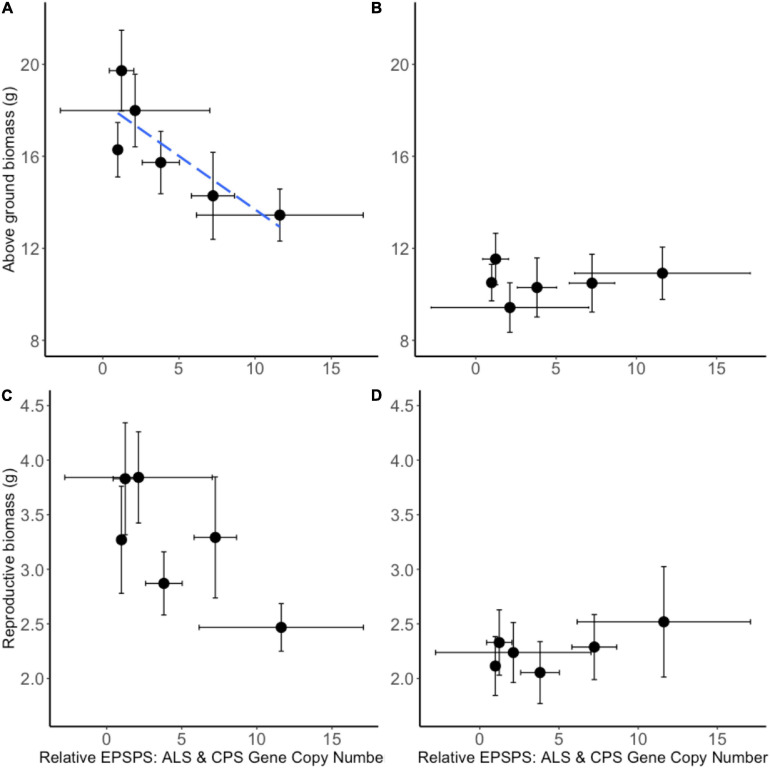
Biomass of common waterhemp seed families grown in neighborhood design experiment. The relationship between *Amaranthus tuberculatus* seed family average above ground biomass **(A,B)** and reproductive biomass **(C,D)** compared to the average 5-enolpyruvylshikimate-3-phosphate synthase (*EPSPS*) gene copy number relative to carbamoyl phosphate synthetase (*CPS*) *and* acetolactate synthase (*ALS*) reference genes. Panels **(A,C)** represent *A. tuberculatus* plant measures when grown under intra-phenotypic competition. Panels **(B,D)** represent *A. tuberculatus* measures when plants are grown in interspecific competition with maize. Ten replicate pots containing four replicate common waterhemp plants were assessed for each seed family and each treatment and 6–8 replicate plants from each seed family were assessed for *EPSPS* gene copy number. The blue dashed line represents a significant linear relationship between variates. A correlation of *r* = –0.83, *p* < 0.05 was found between relative EPSPS gene copy number and above ground biomass **(A)**. A non-significant relationship *r* = –0.76, *p* = 0.08 was between relative EPSPS Gene Copy Number and reproductive biomass **(C)**. No significant relationship was found in the presence of maize **(B,D)**. Error bars are standard errors of the mean.

A neighborhood design competition experiment allowed the study of intraspecific and interspecific resistance costs. *A. tuberculatus* seed families exhibited a continuum of glyphosate resistance levels (and *EPSPS* gene copy numbers), thus making it possible to quantify the trade-off between resistance benefit and resistance cost. Significant negative correlations were observed between above ground biomass in the absence of glyphosate application (a measure of resistance cost) and glyphosate LD_50_ (resistance benefit) (*r* = −0.87, *p* < 0.05; Supplementary Figure 2) and gene copy number (GCN) (*r* = −0.83, *p* < 0.05; Figure 6) illustrating a significant quantitative resistance cost associated with enhanced GCN under intra-phenotypic competition. An additional 20 extra *EPSPS* gene copies (as seen in seed family 1) were associated with a 26.5% reduction in dry biomass. The relationship between reproductive biomass and LD_50_ followed the same trend (*r* = −0.87, *p* < 0.05; Supplementary Figure 2), with a non-significant negative relationship observed between GCN and reproductive biomass (*r* = −0.76, *p* = 0.08; Figure 6). There was no relationship between seed family resistance level and the competitive effect of *A. tuberculatus* on maize and maize had an equal competitive effect on all *A. tuberculatus* seed families. Thus, the fitness cost established by these experiments was not evident under interspecific competition.

## Discussion

The primary mechanism of glyphosate resistance in the *A. tuberculatus* population was gene amplification of the *EPSPS* target site. Seed families with contrasting glyphosate resistance status were successfully generated and higher *EPSPS* gene copy numbers (GCN) were associated with an increase in glyphosate resistance. Higher GCN were also associated with a growth penalty when more resistance *A. tuberculatus* seed families were grown under intra-phenotypic competition. This trade-off was mitigated in the presence of interspecific competition with maize. Further study of the seed families with extreme phenotypes revealed that the most resistant *A. tuberculatus* individuals had a lower competitive effect on neighboring plants than the most susceptible seed family.

### Confirmation of EPSPS Gene Amplification Based Glyphosate Resistance

5-enolpyruvylshikimate-3-phosphate synthase gene amplification has been shown to be the main mechanism of glyphosate resistance found in *A. tuberculatus* populations (Chatham et al., 2015; Kreiner et al., 2019; Murphy et al., 2019). *EPSPS* gene amplification has also been confirmed as the mechanism of evolved glyphosate resistance in *Kochia scoparia*, *Lolium multiflorum* and *Amaranthus spp.* (Gaines et al., 2010; Salas et al., 2012; Nandula et al., 2014; Wiersma et al., 2015). In *A. tuberculatus EPSPS* gene amplification has led to increased *EPSPS* transcripts and increased protein abundance, indicating that the additional gene copies are likely to be functional (Lorentz et al., 2014). Neither target site mutations nor conclusive evidence for the impaired translocation of glyphosate was observed in our study population. Nonetheless, target site mutations and reduced movement of glyphosate have both evolved in *A. tuberculatus* to result in glyphosate resistance (Nandula et al., 2013). *EPSPS* gene amplification represented the main mechanism observed in our glyphosate resistant *A. tuberculatus* population and we found these copies led to an increase in resistance explaining 85% of the observed variation. The quantitative nature of the resistance mechanism has allowed the generation of a genotypic spectrum of seed families to allow a comprehensive assessment of the relationship between gene copy number and fitness.

The multiple *EPSPS* genes in *A. tuberculatus* have been physically mapped to discrete gene clusters on two homologous chromosomes within pericentromeric regions (Dillon et al., 2017). The localization of these genes to pericentromeric regions indicates transposon-mediated local tandem gene duplication (Slotkin and Martienssen, 2007; Tamaru, 2010; Dillon et al., 2017; Gaines et al., 2019). By contrast, multiple gene copies of *EPSPS* in *A. palmeri* have arisen through extrachromosomal circular DNA (eccDNA) structures (Koo et al., 2018). Interspecies hybridization between *A. palmeri* to *A. tuberculatus* is one potential avenue for the transmission of the gene amplification resistance mechanism (Dillon et al., 2017). Indeed, hybridization within the Amaranthus genus has resulted in such transmissions (Nandula et al., 2014; Oliveira, 2017). However, all of the evidence to date confirms that the eccDNA mechanism of gene duplication in *A. palmeri* is independent from the tandem gene duplication observed in *A. tuberculatus* (Gaines et al., 2019). Thus, interspecies hybridization has not transferred the mechanism between the two species, but instead convergent evolution has produced two different mechanisms of gene amplification to combat the same selection pressure. This finding highlights the importance of different mechanisms of rapid evolution within plants to overcome biotic and abiotic stresses (Patterson et al., 2018).

### Resistant Individuals Exhibit a Reduced Competitive Effect

The comparison of inter-phenotypic and intra-phenotypic competition between resistant and susceptible seed families was achieved through a response surface experiment. Susceptible *A. tuberculatus* individuals exhibited a greater competitive effect than resistant individuals, this manifests as increased competitive suppression of neighboring plants across different densities and proportions. A greater competitive effect indicates that resources are allocated to interference competition, impacting neighbors’ ability to acquire resources. Our results illustrate that the ability to suppress the capture of resources by neighboring plants is an effective competitive strategy under intraspecific competition. These findings support the results observed in the neighborhood design experiments. Similar studies investigating herbicide resistant *Lolium rigidum* have found no differences (Pedersen et al., 2007) and extensive costs (Vila-Aiub et al., 2009a) in competitive parameters.

### Intraspecific Competition Found to Produce a Resistance Penalty

We have, for the first time, been able to demonstrate a quantitative trade-off between the resistance benefit (seed family LD_50_) and resistance cost (growth under intraspecific competition in the absence of glyphosate) in a glyphosate resistant weed population with elevated *EPSPS* gene copy number. This work is novel as it explicitly demonstrates that the extent of the trade-off is proportional to the resistance benefit (and the extent of gene amplification), where most other studies have only sought to establish the presence of a qualitative (or semi-quantitative) trade-off. Our results do, however, corroborate other studies which employ alternative methods to elucidate putative fitness costs. Specifically, Wu et al. (2018) grew populations of *A. tuberculatus* under fallow-like settings in the absence of selection and genotype frequencies were observed over six generations. Under these conditions a 10-fold reduction was observed in the number of individuals harboring multiple *EPSPS* gene copies. By contrast, in the same study, the *EPSPS* target site mutation, which also endowed glyphosate resistance, was found to persist where gene amplification declined (Wu et al., 2018). Thus, it appears that the *EPSPS* gene amplification mechanism is commonly associated with a penalty in *A. tuberculatus*. As our experiments were conducted in a single, semi-controlled environment the observed fitness penalties may not translate directly into the field. Indeed, GxE interactions alongside factors such as spatial diversity and crop management can influence the expression and magnitude of fitness costs (Vila-Aiub et al., 2009b). It should be noted that between 6 and 8 individuals were used to calculate average *EPSPS* GCN per seed family, this level of replication in combination with the large GCN variance observed within the families represents a limitation of this study.

Glyphosate resistant *Bassia scoparia* has also been found to contain amplified *EPSPS* gene copies numbers *via* tandem gene duplication (Jugulam et al., 2014). In some genetic backgrounds, these resistant *B. scoparia* individuals were found to exhibit reduced competitive ability and delayed flower emergence (Martin et al., 2017). Furthermore resistant *B. scoparia* was seen to exhibit lower fitness, through reduced seed longevity and seed germination (Osipitan and Dille, 2017). Likewise, glyphosate resistant *Lolium perenne* and *Lolium multiflorum* have also been found to contain *EPSPS* tandem gene amplification, and in this case resistant *L. perenne* produced substantially fewer seeds than susceptible counterparts (Yanniccari et al., 2016). Glyphosate resistant *L. multiflorum* produced lower seed numbers which exhibited lower germination rates (Fernández-Moreno et al., 2017).

It may be hypothesized that the costs observed in this study result from the diversion of resources toward the over-production of *EPSPS*, as suggested in Tang and Amon (2013). Alternatively, if the transposon-mediated local tandem gene duplication is still active within resistant population these selfish elements may disrupt native genes leading to reduced fitness (Dillon et al., 2017). It is also possible that the protective mechanisms which lead to epigenetic silencing of transposons have inadvertently repressed neighboring genes, indeed this phenomenon has been observed across multiple kingdoms and has been found to lead to a reduction in “host” fitness (Choi and Lee, 2020). Interestingly, no trade-off was associated with amplified *EPSPS* gene copy numbers in *A. palmeri* plants grown at two different densities (Vila-Aiub et al., 2014). However, glyphosate resistant palmer amaranth populations were seen to have a lower competitive ability across four crops (Chandi et al., 2013). As mentioned above *A. palmeri* contains an eccDNA mediated mechanism of gene amplification rather than tandem gene duplication. It is clear that the presence of an *EPSPS* gene amplification resistance cost may be influenced by weed species and the genomic mechanism that has mediated an increase in gene copy numbers.

### Interspecific Competition Was Able to Mitigate the Intraspecific Resistance Penalty

The resistance penalty we observed under conditions of intra-phenotypic competition was not evident in the presence of interspecific competition with maize. This finding indicates that growth penalties and potential associated fitness costs are environment specific (there is a genotype by environment interaction at play in determining potential costs of glyphosate resistance). It is not the first time that such a phenomenon has been observed, where the presence of a crop influences the expression of a cost. When glyphosate resistant *Lolium rigidum* was grown in isolation or under low wheat competition, a fitness cost was observed resulting in a 7.5 % reduction in seed production. However, under high crop densities, the fitness cost was mitigated (Pedersen et al., 2007). Subsequent studies showed that the cost observed under low densities was maintained in the field under the absence of selection, revealing a 34% reduction in the resistant phenotype over 3 years (Preston and Wakelin, 2008). The dominance and suppressor model of competition states that size differences are exacerbated under competition (Harper, 1977; Weiner, 1985). Here a larger plant, or one with a faster growth rate, was able to seize the majority of resources resulting in a greater ability to suppress the growth of competitors (Weiner, 1985). We hypothesize that the variance in the exponential growth rates between maize and *A. tuberculatus* contributed to asymmetrical capture of resources resulting in size hierarchies (Schwinning and Weiner, 1998). Such hierarchies have been found to result in increased size inequality at higher densities (Weiner, 1985). Indeed the development of size hierarchies at high plant densities can be explained by unequal light interception (Schwinning and Weiner, 1998) and also differences in growth rate (Schmitt et al., 1986). It is possible that hierarchies may have resulted in partial size asymmetry whereby the maize received a disproportionate share of the resources (Schwinning and Weiner, 1998) thus uniformly restricting the growth of *A. tuberculatu*s. Furthermore, the dominance / suppressor model comes into play at low light levels (Schmitt et al., 1986), and the polytunnel growth environment did not supply additional light, thus potentially assisting maize to act as the dominant competitive suppressor species.

### Exploitation of Fitness Penalties

Knowledge of the presence of fitness trade-offs associated with resistance can be used to design management practices that will reduce the frequency of evolved herbicide resistance (Vila-Aiub, 2019). Mathematical modeling to predict the evolution and spread of resistance can be used to optimize and validate management practices that mitigate the risk of an herbicide resistance epidemic (Vila-Aiub et al., 2005b). Modeling the impact of different management strategies on the evolution of glyphosate resistance in *Amaranthus spp.* has been used to predict resistant genotype frequency over time (Neve et al., 2011; Liu et al., 2020). In the absence of detailed knowledge of the fitness costs associated with glyphosate resistance, these models have made the conservative assumption that there was no cost of resistance. Further study of seed viability and additional reproductive metrics within the study population could provide evidence for the potential to exploit a trade-off associated with amplified *EPSPS* GCN mediated glyphosate resistance in *A. tuberculatus*. Increasing knowledge of the presence and size of fitness costs will enhance efforts to model weed management strategies that mitigate evolution of glyphosate resistance.

## Conclusion

In a series of experiments to explore the fitness costs and benefits of a glyphosate resistance mechanism endowed by amplified gene copy number of the glyphosate target gene, *EPSPS*, we have demonstrated a reduced competitive effect of resistant seed families and a negative trade-off between resistance cost and benefit under conditions of intra-specific competition. There appears to be a genotype by environment component to these trade-offs as they were not observed during inter-specific competition with maize. Costs of glyphosate resistance will moderate and mitigate the rates of evolution of glyphosate resistance and potential selection against resistance in the absence of glyphosate, they are unlikely, however, to lead to significant reductions in the frequency of glyphosate resistance, even with relaxed selection.

## Data Availability Statement

The raw data supporting the conclusions of this article will be made available by the authors, without undue reservation.

## Author Contributions

PN, SK, and HC: conception and design of project. HC and PN: writing manuscript. HC: conducting phenotypic screens, dose response experiments, translocation assessment, competition experiments, and statistical analysis. SH: dose response advice. AH: radio labeling tutoring. LN, HC, and RD: DNA extraction and Q-PCR. All authors contributed to the article and approved the submitted version.

## Conflict of Interest

SK, AH, RD, and SH were employed by the company Syngenta. The remaining authors declare that the research was conducted in the absence of any commercial or financial relationships that could be construed as a potential conflict of interest.

## Supplementary Material

Supplementary Figure 1 Glyphosate resistance mechanism of resistance in common waterhemp **(A)** ^14^C labeled glyphosate translocated away from the site of application in the resistant (R) and susceptible (S) *A. tuberculatus* plants relative to the ^14^C glyphosate recovery at time point zero (T0) **(B)** The proportions of ^14^C labeled glyphosate translocated away from the site of application to the root, stem, rest of the foliage and meristem, relative to the ^14^C glyphosate recovery at time point zero (T0). 12 replicate plants were used per treatment. No significant difference was observed between levels of translocation in R and S plants.

Supplementary Figure 2 Biomass of common waterhemp seed families grown in neighborhood design experiment. The relationship between *Amaranthus tuberculatus* seed family average above ground biomass **(A,B)** and reproductive biomass **(C,D)** compared to the resistance level glyphosate lethal dose required to kill 50 % of individuals (LD_50_). Panels **(A,C)** represent *A. tuberculatus* plant measures when grown under intra-phenotypic competition. Panels **(B,D)** represent *A. tuberculatus* measures when plants are grown in interspecific competition with maize. The LD_50_ for each seed family was calculated using 30 replicate plants for each of six glyphosate doses. Ten replicate pots containing four replicate common waterhemp plants were assessed for each seed family and each treatment. The blue dashed lines represent significant linear relationships between variates. A correlation of *r* = −0.87, *p* < 0.05 was found between seed family LD_50_ and above ground biomass **(A)** and a correlation of *r* = −0.87, *p* < 0.05 was found between seed family LD_50_ and reproductive biomass **(C)**. Error bars are standard errors of the mean.

Supplementary Table 1 Primer sequences for qPCR.

Supplementary Table 2 *p* values for LD_50_ value student *t*-test comparison between each seed family. Bold text indicates a non-significant difference between seed families. Families are ordered left to right from most susceptible to most resistant.

## Abbreviations

ALS: acetolactate synthase
CPS: carbamoyl phosphate synthetase
Δ CT: change in cycle threshold
eccDNA: extrachromosomal circular DNA
*EPSPS*: 5-enolpyruvylshikimate-3-phosphate synthase
GCN: Gene copy number
LD_50_: lethal dose 50
R: resistant
RI: Resistance Index
S: Susceptible
TE: transposable element
qPCR: quantitative polymerase chain reaction.
