# Supplementary figures and images for: Fitness Cost Associated With Enhanced EPSPS Gene Copy Number and Glyphosate Resistance in an Amaranthus tuberculatus Population
Source: Front Plant Sci. 2021 Jun 29;12:651381. doi: 10.3389/fpls.2021.651381 (PMC8276266; doi:10.3389/fpls.2021.651381)

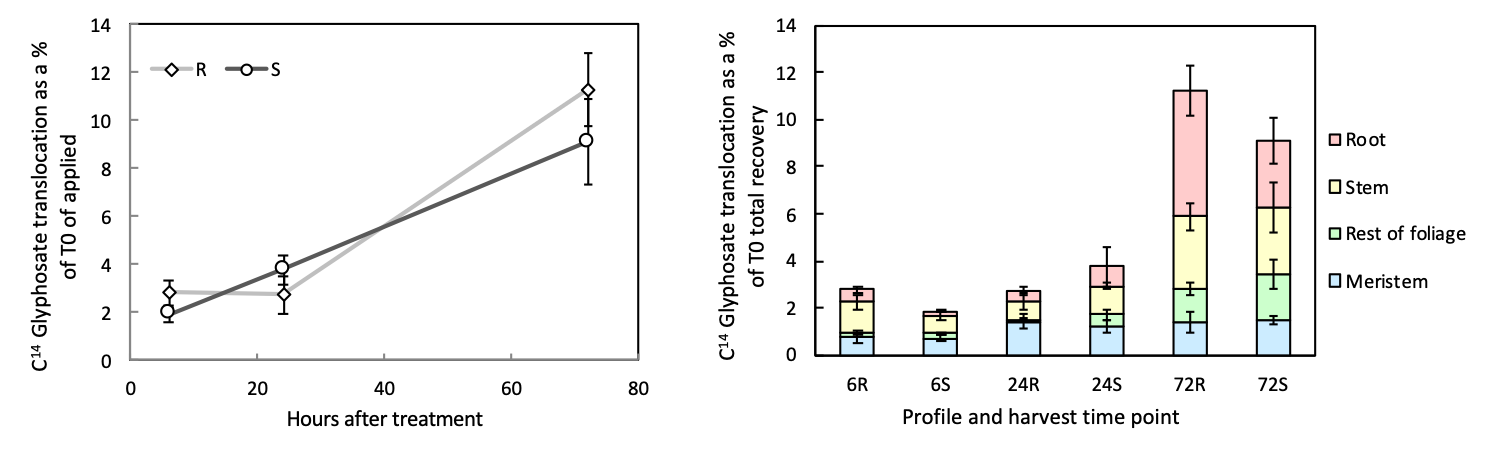

Supplement: Supplementary Figure 1 — Glyphosate resistance mechanism of resistance in common waterhemp (A) 14C labeled glyphosate translocated away from the site of application in the resistant (R) and susceptible (S) A. tuberculatus plants relative to the 14C glyphosate recovery at time point zero (T0) (B) The proportions of 14C labeled glyphosate translocated away from the site of application to the root, stem, rest of the foliage and meristem, relative to the 14C glyphosate recovery at time point zero (T0). 12 replicate plants were used per treatment. No significant difference was observed between levels of translocation in R and S plants. [file Image_1.TIFF]

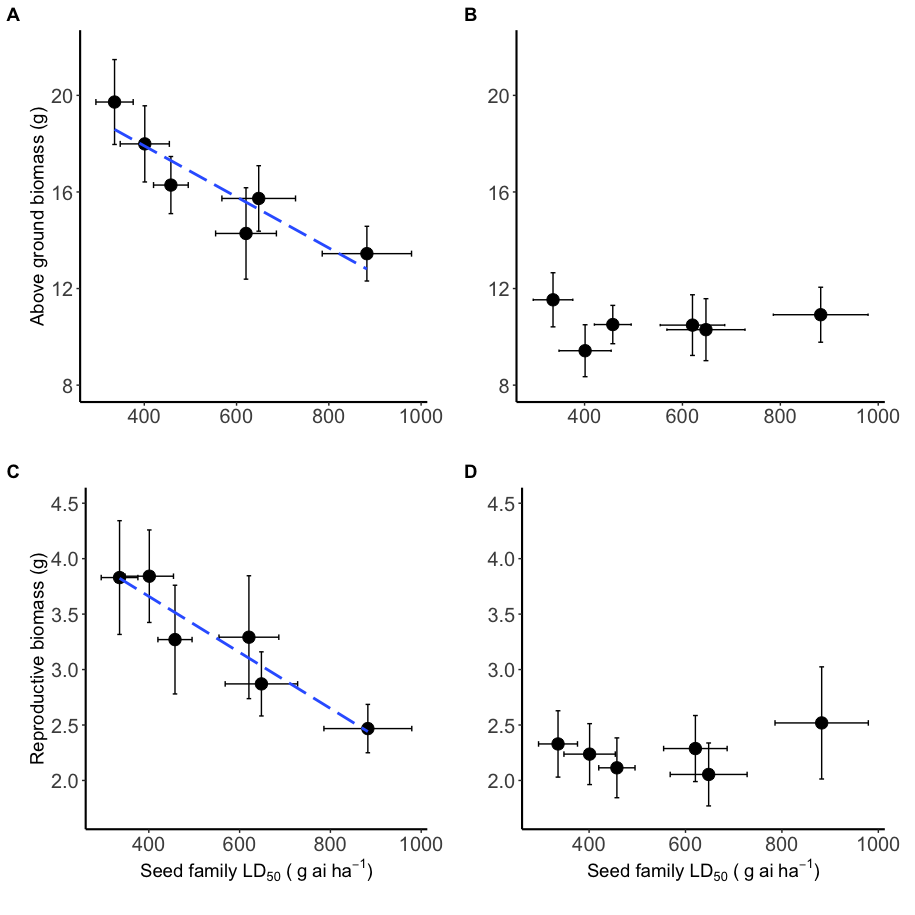

Supplement: Supplementary Figure 2 — Biomass of common waterhemp seed families grown in neighborhood design experiment. The relationship between Amaranthus tuberculatus seed family average above ground biomass (A,B) and reproductive biomass (C,D) compared to the resistance level glyphosate lethal dose required to kill 50 % of individuals (LD50). Panels (A,C) represent A. tuberculatus plant measures when grown under intra-phenotypic competition. Panels (B,D) represent A. tuberculatus measures when plants are grown in interspecific competition with maize. The LD50 for each seed family was calculated using 30 replicate plants for each of six glyphosate doses. Ten replicate pots containing four replicate common waterhemp plants were assessed for each seed family and each treatment. The blue dashed lines represent significant linear relationships between variates. A correlation of r = −0.87, p < 0.05 was found between seed family LD50 and above ground biomass (A) and a correlation of r = −0.87, p < 0.05 was found between seed family LD50 and reproductive biomass (C). Error bars are standard errors of the mean. [file Image_2.tiff]
